# Supplementary material for: Natural Course of IQSEC2-Related Encephalopathy: An Italian National Structured Survey
Source: Children (Basel). 2023 Aug 24;10(9):1442. doi: 10.3390/children10091442 (PMC10528631; doi:10.3390/children10091442)
Supplement: Supplementary file 1 [file children-10-01442-s001.zip › Supplementary Table S1.pdf]

Supplementary Table S1. Prevalence estimates of *IQSEC2*-related encephalopathy for the Italian population

Patient ID #	Gender	Age (years)	Age class for age pyramid[1]	*n* for same-age class and gender[1]	Prevalence x 10^{-7}
1	M	16.7	0 to 4	1 101 059	9.08217
2	F	22	20 to 24	1 390 957	7.18929
3	F	19.4	15 to 19	1 384 080	7.22502
4	M	19.8	15 to 19	1 482 037	6.74747
5	M	15.5	15 to 19	1 482 037	6.74747
6	F	11.6	10 to 14	1 369 852	7.30006
7	M	12.7	10 to 14	1 453 792	6.87856
8	F	15.7	15 to 19	1 384 080	7.22502
9	F	12.6	10 to 14	1 369 852	7.30006
10	F	10.3	10 to 14	1 369 852	7.30006
11	F	12.1	10 to 14	1 369 852	7.30006
12	F	34.0	30 to 34	1 580 224	6.32822
13	M	2.7	0 to 4	1 101 059	9.08217
14	M	3.5	0 to 4	1 101 059	9.08217
15	M	8.5	5 to 9	1 297 135	7.7093
16	F	13.6	10 to 14	1 369 852	7.30006
17	M	15.0	15 to 19	1 384 080	7.225
18	M	2.3	0 to 4	1 101 059	9.08217
19	F	12.5	10 to 14	1 369 852	7.30006

[1] Data extracted from updated national demographic report [63]
