# Supplementary material for: Natural Course of IQSEC2-Related Encephalopathy: An Italian National Structured Survey
Source: Children (Basel). 2023 Aug 24;10(9):1442. doi: 10.3390/children10091442 (PMC10528631; doi:10.3390/children10091442)
Supplement: Supplementary file 1 [file children-10-01442-s001.zip › Supplementary Table S2.pdf]

Supplementary Table S2. *IQSEC2* variants, variants in other gene/s, and/or rearrangements in the Italian *IQSEC2*-population.

Patient ID #	Gender	IQSEC2 variant	Inheritance	*IQSEC2* Mutation type	ACMG categorization ClinVar	Variants/ rearrangements	Variant/ rearrangemen inheritance[1]
1	M	c.2369G>A p. Arg790Gln	Mat.	Missense	Pathogenic (PP5 PM2 PM5 PM1 PP3 PP2) ClinVar (Accession VCV000280590.13)	*SMARCC1* (c.644delA)	*de novo*
2	F	c.2369G>A p. Arg790Gln	Mat.	Missense	Pathogenic (PP5 PM2 PM5 PM1 PP3 PP2) ClinVar (Accession VCV000280590.13)	–	–
3	F	c.3781C>T p. Gln1261*	NA	Nonsense	Likely Pathogenic (PVS1 PM2) ClinVar NR	*ANK3* (c.671C>T)	NA
4	M	c.1865_1871dup p. Asp624Glnfs*3	*de novo*	Frameshift	Likely Pathogenic (PVS1 PM2) ClinVar NR	–	–
5	M	c.2750-2A>G	*de novo*	Splicing	Likely Pathogenic (PVS1 PM2) ClinVar NR	–	–
6	F	c.3613_3613delC p. Leu1205Trpfs*192	*de novo*	Frameshift	Likely Pathogenic (PVS1 PM2 PP5) ClinVar (Accession VCV001802601.1)	–	–
7	M	c.854del p. Pro285Leufs*21	*de novo*	Frameshift	Pathogenic (PVS1 PM2 PP5) ClinVar (Accession VCV000619985.6)	–	–
8	F	c.3011T>C p.Leu1004Pro	*de novo*	Missense	VUS (PM2 PP3 PM1 PP2) ClinVar NR	–	–
9	F	c.4039dupG p. Ala1347Glyfs*40	*de novo*	Frameshift	Pathogenic (PVS1 PP5 PM2) ClinVar (Accession VCV000422032.7)	–	–
10	F	c.267C>G p. Tyr89*	*de novo*	Nonsense	Likely Pathogenic (PVS1 PM2) ClinVar NR	Dup7p21.3 (12,728,851x2,12,756,913-13,374,587x2)	Pat.
11	F	c.4110_4111del p. Tyr1371Glnfs*15	*de novo*	Frameshift	Likely Pathogenic (PVS1 PM2) ClinVar NR	–	–
12	F	c.2459+1G>A	*de novo*	Splicing	Likely Pathogenic (PVS1 PM2) ClinVar NR	*MMACHC* (c.331C>T)<br>*COL4A2* (c.2460G>C)<br>*MMACHC* (c.742T>C)	Pat.<br>Pat.<br>Mat.
13	M	c.2459+1G>A	Mat.	Splicing	Pathogenic (PVS1 PM2) ClinVar NR	*TGFB1* (c.551T>C)	Pat.
14	M	c.944_945insGAGGAGGAGATAAAGCG p. Ser316Argfs*4	Mat.	Frameshift	Likely Pathogenic (PVS1 PM2) ClinVar NR	–	–
15	M	c.2488_2490delTCC p. Ser830del	Mat.	In-frame deletion	VUS (PM2 PM4) ClinVar NR	Del4q28.3 (138,062,769 – 138,259,510)<br>DupXp22.33/Yp11.32(chrX:970,499-1,118,325, chrY:920,499-1,068,325)	NA<br>Pat.
16	F	c.2272C>T p. Arg758*	*de novo*	Nonsense	Pathogenic (PVS1 PM2) ClinVar NR	–	–
17	M	c.2225G>A p. Trp742*	*de novo*	Nonsense	Pathogenic (PVS1 PM2 PP5) ClinVar (Accession VCV001323121.2)	*RELN* (c.5156C>T)<br>*GLI3* (c.2424A>G)<br>*GLDC* (c.2113G>A)	NA<br>Pat.

						ARFGEF2 (c.4462A>T)	NA<br>NA
18	M	c.2507C>G<br>p. Ala836Val	NA	Missense	Likely pathogenic (PP3 PM2 PM5 PP2)<br>ClinVar NR	*ATP13A2* (c.477+2T>G)<br>*PEX12* (c.987_988del)	NA<br>NA
19	F	c.2890-2A>G	*de novo*	Splicing	Likely pathogenic (PVS1 PM2)<br>ClinVar NR	–	–

[1] Legend: Mat.=maternally inherited, Pat.=paternally inherited, NA=not available. From ACMG and ClinVar databases [48,49]. VUS=variant of uncertain significance, NR= not reported.
