# Supplementary material for: Natural Course of IQSEC2-Related Encephalopathy: An Italian National Structured Survey
Source: Children (Basel). 2023 Aug 24;10(9):1442. doi: 10.3390/children10091442 (PMC10528631; doi:10.3390/children10091442)
Supplement: Supplementary file 1 [file children-10-01442-s001.zip › Supplementary Table S3.pdf]

Supplementary Table S3. *IQSEC2* variants, mutation type, and illness severity in the Italian *IQSEC2*-population.

Patient ID #	Age (years)	Gender	IQSEC2 variant	Inheritance[1]	*IQSEC2* Mutation type	Phenotypical severity						
						CSS	MBAS	RSBQ	GHQ	Bristol stool form scale	SDSC	QI-Disability
1	16.7	M	c.2369G>A p. Arg790Gln	Mat.	Missense	19	51	35	NA	1	39	78
2	22.0	F	c.2369G>A p. Arg790Gln	Mat.	Missense	6	30	14	NA	4	29	67
3	19.4	F	c.3781C>T p. Gln1261*	NA	Nonsense	8.5	20	26	4	4	40	101
4	19.7	M	c.1865_1871dup p. Asp624Glnfs*3	*de novo*	Frameshift	27.5	58	48	12	4	56	74
5	15.5	M	c.2750-2A>G	*de novo*	Splicing	25	34	27	7	2	31	68
6	11.6	F	c.3613_3613delC p. Leu1205Trpfs*192	*de novo*	Frameshift	9.5	28	26	4	3	40	65
7	12.7	M	c.854del p. Pro285Leufs*21	*de novo*	Frameshift	22	41	31	7	2	41	92
8	15.7	F	c.3011T>C p.Leu1004Pro	*de novo*	Missense	16.5	61	25	9	4	55	87
9	12.6	F	c.4039dupG p. Ala1347Glyfs*40	*de novo*	Frameshift	17	50	57	2	4	47	125
10	10.3	F	c.267C>G p. Tyr89*	*de novo*	Nonsense	6.5	18	14	7	1	32	65
11	12.1	F	c.4110_4111del p. Tyr1371Glnfs*15	*de novo*	Frameshift	20	31	42	8	1	38	67
12	34	F	c.2459+1G>A	*de novo*	Splicing	7	26.5	17	3	6	32	77
13	2.7	M	c.2459+1G>A	Mat.	Splicing	30	54	25	8	1	39	68
14	3.5	M	c.944_945insGAGGAGGAGATAAAGCG p. Ser316Argfs*4	Mat.	Frameshift	29	44	33	6	1	60	93
15	8.4	M	c.2488_2490delTCC p. Ser830del	Mat.	In-frame deletion	5.5	16	16	2	4	32	72
16	13.7	F	c.2272C>T p. Arg758*	*de novo*	Nonsense	12	44	34	5	4	49	73
17	15	M	c.2225G>A p. Trp742*	*de novo*	Nonsense	32.5	62	55	5	4	52	79
18	2.3	M	c.2507C>G p. Ala836Val	NA	Missense	19	21	27	1	9	39	60
19	12.5	F	c.2890-2A>G	*de novo*	Splicing	6	35	12	0	2	57	86

[1] Legend: Mat.=maternally inherited, Pat.=paternally inherited. [2] From ACMG and ClinVar databases [48,49]. CSS=Rett Clinical Severity Score [53], MBAS=Motor Behavior Assessment Scale [54], RSBQ=Rett Syndrome Behavior Scale [55], Gastrointestinal Health Questionnaire (GHQ) [56], SDSC=Sleep Disturbance Scale for Children Questionnaire (SDSC) [58], NA= not available.
